# Precise strategies for selecting probiotic bacteria in treatment of intestinal bacterial dysfunctional diseases

**DOI:** 10.3389/fimmu.2022.1034727

**Published:** 2022-10-20

**Authors:** Jiajun Yang, Shunyi Qin, Hao Zhang

**Affiliations:** ^1^ School of Animal Husbandry and Veterinary Medicine, Jiangsu Vocational College of Agriculture and Forestry, Jurong, China; ^2^ College of Animal Science and Technology, Chinese Agricultural University, Beijing, China; ^3^ Key Laboratory of Agricultural Animal Breeding and Healthy Breeding of Tianjin, College of Animal Science and Veterinary Medicine, Tianjin Agricultural University, Tianjin, China

**Keywords:** probiotics, core bacteria, precise supplementation, target isolation, gut abnormalities

## Abstract

Abundant microbiota resides in the organs of the body, which utilize the nutrition and form a reciprocal relationship with the host. The composition of these microbiota changes under different pathological conditions, particularly in response to stress and digestive diseases, making the microbial composition and health of the hosts body interdependent. Probiotics are living microorganisms that have demonstrated beneficial effects on physical health and as such are used as supplements to ameliorate symptoms of various digestive diseases by optimizing microbial composition of the gut and restore digestive balance. However, the supplementary effect does not achieve the expected result. Therefore, a targeted screening strategy on probiotic bacteria is crucial, owing to the presence of several bacterial strains. Core bacteria work effectively in maintaining microbiological homeostasis and stabilization in the gastrointestinal tract. Some of the core bacteria can be inherited and acquired from maternal pregnancy and delivery; others can be acquired from contact with the mother, feces, and the environment. Knowing the genera and functions of the core bacteria could be vital in the isolation and selection of probiotic bacteria for supplementation. In addition, other supporting strains of probiotic bacteria are also needed. A comprehensive strategy for mining both core and supporting bacteria before its clinical use is needed. Using metagenomics or other methods of estimation to discern the typically differentiated strains of bacteria is another important strategy to treat dysbiosis. Hence, these two factors are significant to carry out targeted isolation and selection of the functional strains to compose the resulting probiotic preparation for application in both research and clinical use. In conclusion, precise probiotic supplementation, by screening abundant strains of bacteria and isolating specific probiotic strains, could rapidly establish the core microbiota needed to confer resilience, particularly in bacterial dysfunctional diseases. This approach can help identify distinct bacteria which can be used to improve supplementation therapies.

## 1 Introduction

Probiotics are live bacteria that confer health benefits to the host when administered in adequate quantities (1). Recent studies have demonstrated that the host’s microbiota plays an important role in maintaining overall health, and is thus an attractive target for clinical interventions (2, 3). In humans and animals, the gastrointestinal tract (GIT) contains a dense population of microorganisms that cohesively interplay with the host’s digestion and help to fight against infections (2, 4). Some of the bacterial strains present belong to core genera that may represent a significant proportion of the GIT microbiota or form smaller dominant microbial groups (5). However, many factors, such as alcohol consumption and high-energy diets containing lots of carbohydrates and proteins, can cause dysbiosis of the GIT (6). Significant consequences of nutritional and metabolic diseases are disturbance of the GIT microbiota, especially under stress conditions (7, 8). Treat these diseases involves restoring the microecological environment of the GIT. Thus, as an alternative to administering antibiotic drugs, supplementation with probiotic bacteria has been found to be an effective approach. Scientists have developed a growing interest in assessing the ability of probiotics to enhance the health of humans or animals experiencing microbial dysbiosis (9). Numerous strains of bacteria, from both humans and animals, have been isolated from different organs (10). Furthermore, these isolated probiotic bacteria have been used in products designed and developed for clinical use, both in human medicine and animal husbandry and breeding (6, 11).

Currently the most widely used probiotics such as *Bifidobacteria*, *Propionibacteria*, *Lactobacillus*, *Bacillus*, *Akkermansia muciniphila*, and *Saccharomyces* play important roles when used and administrated in certain strains and approaches. Supplementing these probiotics can help modulate the GIT microbiota, thereby alleviating symptoms of inflammatory bowel diseases. The myriad of benefits associated with probiotic supplementation are shown in **Table 1** (12–27). However, it is unclear if probiotic bacteria containing products are effective in treating all cases of dysbacteriosis or if they can function as a nutritional supplement (28). The probiotic bacteria used in clinical did not realize the respected aims even meaningless (29–31). Optimal methods of isolating and identifying suitable probiotic bacterial strains for use in humans and animals are yet to be determined.

**Table 1:** Examples of beneficial effects of probiotics on humans and animal models.

Probiotic bacteria	Health effect	Models	References
*Bifidobacteria*: *B*. *infantis*, *B*. *longum B*. *animalis*, *B*. *pseudocatenulatum*	Ulcerative colitis, Crohn’s disease (CD), Ameliorate inflammation caused by gliadin, Reduce IBS symptoms, Reduce plasma C-reactive protein and IL-6 in UC Control metabolic disorders, Improved glucose tolerance	Human, Rat	12–15.
*Propionibacteria*: *P*. *freudenreichii*, *P*. *jensenii* 702, *P*. *acidipropionici*	Clostridium difficile infection (CDI), Immunomodulation, Microbiota modulation, Binding of toxic compounds, Diarrhea	Human, Mice	16–18
*Lactobacillus*: *L*. *plantarum*, *L*. *acidophilus*, *L*. *salivarius*, *L*. *reuteri*, *L*. *bulgaricus*	Microbiota modulation, Inhibition the colonization of pathogen,	Human, Mice, Poultry, Calves, Swine.	2, 19, 20
*Bacillus*: *B*. *subtilis*, *B*. *icheniformis*, *B*. *coagulans*, *B*. *egaterium*	Secretion of digestive enzymes, antimicrobials, vitamins & elements.	Human, Poultry, Calves, Swine	6, 21, 22
*Akkermansia muciniphila*	Degrade mucin, synthesize multiple amino acids, vitamins, and cofactors, Immunomodulation.	Human, Mice.	9, 23.
*Saccharomyces*: *S. boulardii*, *S*. *cerevisiae* *S*. *boulardii*.	Treatment of diarrhea, inflammatory bowel diseases, antibacterial action on *Salmonella* enterica serovar *Typhimurium* or *Clostridium difficile*.	Human, Mice.	24–26
*Pseudomonas*, *Staphylococcus*, *Acinetobacter*	Existing in colostrum, help to establish core bacteria for neonate	Human, Cow.	

The review discusses the intrinsic roles of bacteria in the GIT and typical microbial differences observed in digestive diseases and stress-induced micro dysbiosis. Identifying the unique strains of bacteria associated with intestinal microbial dysfunctional diseases is greatly important for developing effective treatments for the disease. This review also aims to discuss strategies for the precise choice of probiotic bacteria based on how the host’s microbiota needs to be modulated to restore balance, to provide researchers with effective methods to isolate probiotic bacteria and reduce candidate strains to achieve effective targeting and precise supplementation for clinical applications.

## 2 Causal relationship between dysbacteriosis and diseases

The gut microbiota consists of trillions of microbial cells belonging to many different strains of bacteria. This complex group of microorganisms is established shortly after birth and is subsequently influenced by factors such as diet, geography, genetics, medications, and lifestyle (32). Since the GIT is the site of digestion and absorption of nutrients, the microbial composition is primarily influenced by nutritional changes (33). Under homeostatic conditions, the gut microbiota is in a reciprocal relationship with the host and has important roles in maintaining health in relation to food metabolism, pathogen defense, immune training, production of important metabolites, and neuro-endocrine regulation (34). Most of the constituent strains are the same across different healthy individuals. The diversity and similarity in microbial composition of the GIT can both be caused by or result in digestive disease, especially under stress conditions (8).

Nutritional and metabolic disease threatens human health, and are therefore a focus of studies on the relationship between altered microbial composition and poor physical health (35, 36). Nutritional and metabolic diseases include obesity, gout, and non-alcoholic fatty liver disease. Consuming a diet rich in carbohydrates and proteins usually causes an alteration in the composition of GIT bacteria (37). Obesity is an epidemic phenomenon and is a prime risk factor for type 2 diabetes, gout, and cardiovascular diseases (38). In general, obese individuals show lower microbial diversity and gene richness than do non-obese individuals, and the difference in phylum levels appeared to be significant (39). An increased abundance of the phylum Firmicutes and a decreased abundance of Bacteroidetes have been observed in obese individuals. In mouse models, the caecum was found to normally be dominated by Firmicutes (60–80% of the phylotypes) and Bacteroidetes (20–40% of the phylotypes), but an approximately 50% reduction in the abundance of Bacteroidetes can be observed in obese mice relative to lean mice (40, 41). An increased ratio (5:1 to 6:1) of Firmicutes to Bacteroidetes is regularly be observed in obese humans and animals. According to the conceptual framework of Koch’s postulates, microbial dysbiosis is both a symptom and cause of many diseases (42, 43). Gut microbiota transplantation experiments have revealed a causal correlation between the gut microbiota and the development of obesity and type 2 diabetes (42). When germ-free (GF) mice were colonized with GIT microbiota isolated from obese mice, the GF mice acquired more body fat than mice colonized with microbiota from lean mice, indicating a contributory effect of the microbiota composition to obesity (5, 37).

Obesity, type 2 diabetes, and inflammatory bowel diseases (IBDs), including Crohn’s disease and ulcerative colitis, are chronic inflammatory conditions of the intestinal tract that affect humans and cause significant morbidity and occasional mortality (44, 45). IBD patients tend to have low bacterial diversity as well as lower numbers of Bacteroidetes and Firmicutes. These factors, together, reduced concentrations of microbial-derived butyrate. Butyrate and other short chain fatty acids are thought to have a direct anti-inflammatory effect in the gut (45). IBDs are often accompanied by microbial dysfunction which is not simply caused by a single pathogen. Furthermore, different indices of IBD activities have each been characterized by specific gut mucosa-attached bacteria. Strains of probiotic bacteria have proved effective in reducing IBD symptoms by improving the microbial composition and repairing the mucous membrane (46). However, there are no suitable cures containing probiotics and studies on the use of probiotics in clinical applications have mixed results (47, 48).

## 3 Distinguishing microbial differences associated with diseases

In nutritional and metabolic diseases, significant microbial differences have been observed at the phylum and genus levels. Similar results were observed for colon cancer, obesity, and non-alcoholic fatty liver disease (49).

**Figure 1:**
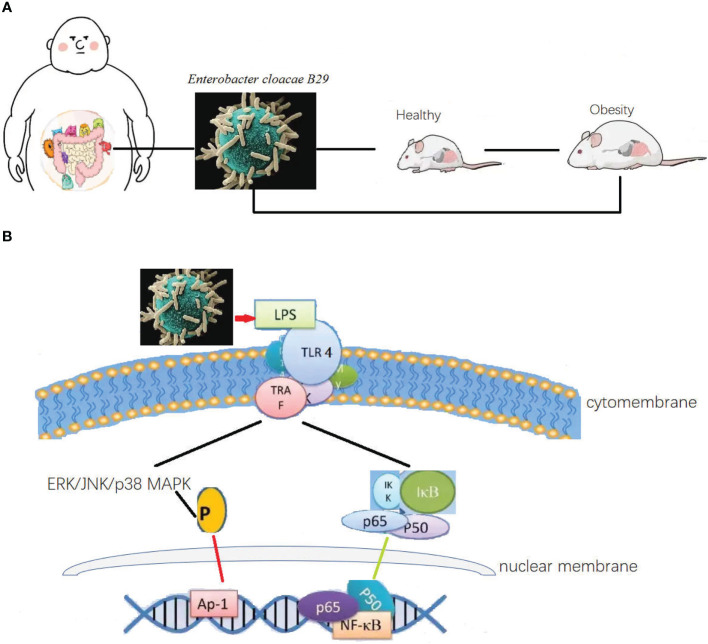
The differential bacteria in obesity and as a causation to obese. **(A)** Intestinal bacteria was both the cause and syndrome for the obesity. Causation relationship between the disease and organic bacteria. Microbiota transplanted from the obesity children to germ free mice caused the same syndromes. The strain of *Enterobacter cloacae B29* was proved as the prime culprit to little fatties’ children syndrome. Toxins such LPS produced through playing pleiotropic role one pathway was recognized by toll like receptor 4 to upstream signal pathway to regulate gut permeability, and control adipose tissue metabolism through mediating cannabinoid-driven adipogenesis, which accelerated the syndrome of obesity. **(B)** Then possible molecular signal pathway of *Enterobacter cloacae B29* regulated the adipose tissue metabolism. The toxin produced by Enterobacter cloacae B29 can promote the syndrome. Toxins such LPS produced through playing pleiotropic role one pathway was recognized by toll like receptor 4 to upstream signal pathway to regulate gut permeability. The signaling pathways involved include the ERK/p38/JNK and nuclear transcription factor pathways were enrolled to control adipose tissue metabolism through mediating cannabinoid-driven adipogenesis, which accelerated the syndrome of obesity.

Especial in little fatties’ children, with some obesity was inheritable, the overweight caused hyperglycemia, hypertension, and dyspnea. This situation has been popular in high carbohydrate contained diet. The fecal transplanted from the obesity children to germ free mice caused the same syndromes (**Figure 1A**). The strain of Enterobacter cloacae B29 was proved as the prime culprit to little fatties’ children syndrome. The toxin produced by *Enterobacter cloacae B29* can promote the syndrome (43). Toxins such LPS produced through playing pleiotropic role one pathway was recognized by toll like receptor 4 (TLR4) (**Figure 1B**) to upstream signal pathway to regulate gut permeability. Classic mitogen-activated protein kinases (MAPK), which were divided into 4 subgroups: ERK/p38/JNK and BMK1 (50). The signaling pathways involved include the ERK/p38/JNK and nuclear transcription factor (NF-kB) pathways were enrolled to control adipose tissue metabolism through mediating cannabinoid-driven adipogenesis, which accelerated the syndrome of obesity (50, 51). Cross talk between the endotoxins produced by the strain and the TLR4 of the host is the most upstream and vital molecular event responsible for inducing all the phenotypes of obesity and other digestive diseases (52–54). Overgrowth in the human gut of these nonvirulent endotoxin-producing strains of pathogenic bacteria species, may collectively become a predictive biomarker or serve as a novel therapeutic target for treatment of obesity, non-alcoholic fatty liver disease, and other related metabolic disorders (55).

The commensal bacterial species *Bacteroides fragilis*, *Fusobacterium nucleatum*, and *Escherichia coli* (*E*. *coli*) seem to emerge as pathogens and contribute to colorectal carcinogenesis through their inflammatory and oncogenic properties (53). Additionally, *Bacteroides fragilis* has been shown to be enriched in the gut microbiota of patients with colorectal cancer (54). Along with an increased abundance of *Bacteroides fragilis*, a decreased population of *Bacteroides vulgatus* and *Bacteroides stercoris* has also been observed in the guts of patients with human colorectal cancer (52, 55, 56). Studies on specific bacterial species associated with obesity and non-alcoholic fatty liver disease, and their molecular cross talk with the host, have suggested that overgrowth of nonvirulent endotoxin-producing strains of pathogenic bacteria, such as *Enterobacter cloacae B29*, *E*. *coli PY102*, and *Klebsiella pneumoniae A7*, in the gut of obese humans can act as causative agents for non-alcoholic fatty liver disease (54).

The microbial composition of the GIT is affected by diet and disease, and is also a typical symptom of many diseases. Further, microbial translocation to the rest of the body can make them causative agents of disease (57). All these results suggest that the microbial composition is not only the causative agent, but also an outcome of the development of various diseases.

Diarrhea and common infectious diseases of the GIT are mainly caused by infection-causing pathogens or endogenous opportunistic pathogens, accompanied with other typical clinical symptoms (58). In patients with diarrhea, the water content of the excreta increases more than that under healthy conditions. The microbial composition is disturbed by endogenous opportunistic pathogens. The functioning of the intestinal barrier is reduced and water filters more readily through the mucous membrane to reach the inner lumen of the intestine. Additionally, an increase in the abundance of endogenous *E*. *coli* or *Salmonella typhi* results in further deterioration of symptoms (59–62). The microbial composition of the gut can be both a causative agent and result of diarrhea and infectious gastrointestinal diseases. Toxins and endotoxins produced by pathogens or overgrowth of opportunistic pathogens act to impair and destroy the mucous membrane through a cascade of molecular signals, which is recognized by TLR4 (63), which presents the signal to MyD88, thereby stimulating the downstream expression of interleukin-1β (IL-1β) and tumor necrosis factor α (TNF-α). The signaling pathways involved include the classic nuclear transcription factor (NF-kB) or c-Jun N-terminal kinase pathways (**Figure 1B**) which act to up-regulate inflammation and suppress the activity of T regulatory cells (Tregs) (64), which were partly agreed with the obesity caused by *Enterobacter cloacae B29*. Under unfavorable environmental conditions, such as high ammonia concentration, heat, cold, and transportation, animals can suffer abnormal bodily stress. Stress can induce alterations in the microbiota of the digestive and respiratory tracts. Furthermore, the altered microbiota can negatively impact the health of the animal through microbial translocation.

## 4 Common supplementary methods of probiotic bacteria

Considering the relationship between organs of the body and microbiota, the restoration of microbiota should be the first step in the treatment of dysbiosis-related diseases. Resilience of dominant bacteria in the GIT of patients can be the most meaningful strategy for in restoring digestive balance (52, 65, 66). Fecal microbiota transplantation (FMT), which involves preparation and administration of distal gut microbiota-containing fecal material from healthy donors to a patient with a disrupted gut microbiota is, a promising strategy in the treatment of microbiota dysbiosis diseases. FMT acts to directly interfere with gut microbiota receptors, thus normalizing the microbial composition and producing therapeutic effects (67, 68). FMT has widely employed since 2013, when the United States Food and Drug Administration approved FMT for treatment of *Clostridium difficile* infection (69, 70). Since then, the range of its applications extended rapidly and broadly, not only for the treatment of gastrointestinal disorders, but also for extra-gastrointestinal disease, such as obesity, type 2 diabetes, and even cancer (71). Patients with Crohn’s disease, obesity, type 2 diabetes, and chronic IBD who received FMT treatment experienced therapeutic benefits (72, 73). Thus, the restoration of the microbial community is important to overcoming disease.

Reduce the over-growth of bacteria and conditioned bacteria, eliminate the pathogen. For the treatment of diarrhea and infectious gastrointestinal diseases, physicians often prescribe tablets containing strains of probiotic bacteria, as an alternative to antibiotic drugs, owing to the numerous side effects of antibiotics, particularly antibiotic resistance. Many strains of drug resistant bacteria, such as carbapenem-resistant *Enterobacteriaceae*, vancomycin-resistant *Enterococcus*, and extended spectrum beta-lactamase carrying strains, represent a major public health concern, as they are potential pathogens associated with a high mortality rate (74). Thus, the common genera of probiotic bacteria, including *Bacillus*, *Bifidobacterium*, *Akkermansia*, and *Clostridium* have often been used to inhibit *E*. *coli* or *Salmonella* growth (71, 75, 76). However, if the microbial composition of the patient is unknown, a generalized use of probiotic bacteria may not provide the desired effect.

An alternative means to prevent diarrhea and improve body health is dietary supplementation with probiotic bacteria or with fermented food containing probiotic bacteria, namely those in the genera *Lactobacillus*, *Bacillus*, *Saccharomyces*, and *Bifidobacterium* (77, 78). Effects of probiotic supplementation include improving intestinal secreting mucins, enhancing mucosal barrier function, increasing tight junctions in mucous cells, providing colonization resistance, increasing production of secretory lgA, producing bacteriocins, producing balanced T-helper cell response, and increasing production of IL-10 and Tregs (79, 80). However, the origin of some used probiotic bacteria was not clear, and the physiological characters of bacteria was lack of sufficient studied. In clinical use, the diagnosis effect maybe off-target and not achieved. Many probiotic bacteria were overused. Analyze the composition of microbiota in GIT of patient, provided the precise supplementary strategies could be needed.

## 5 Precise supplementary strategies

### 5.1 Core bacteria and its function

**Figure 2:**
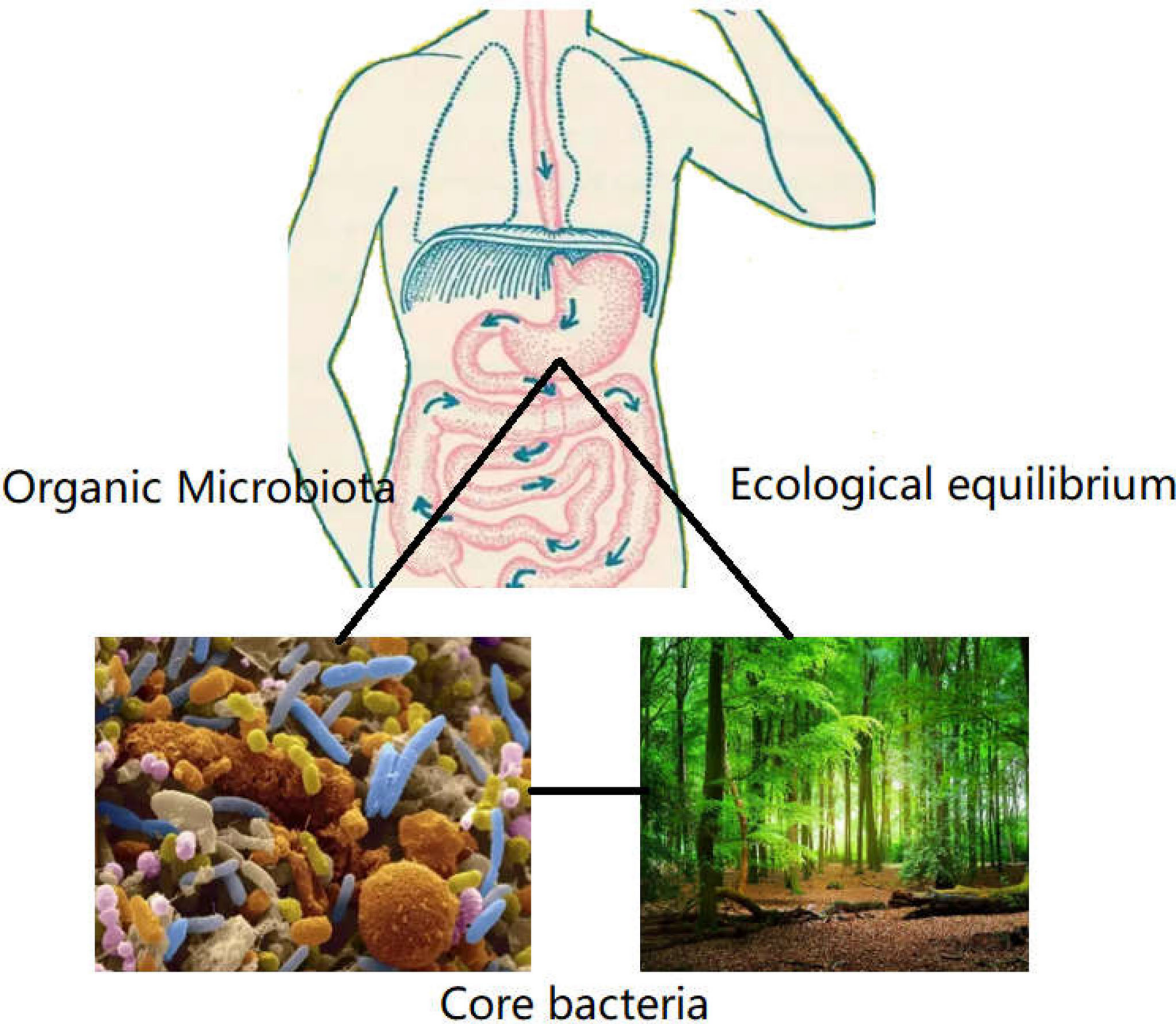
The role of core bacteria in organs. Organs such as mouth, gastrointestinal tract, lung, and genitourinary tract harbored many strains of bacteria, like the virgin forest existing many floras. The core bacteria were the exuberant floras, which presided in the microbiome. Although the number of core bacteria covered a few proportions in total flora. The function was the biggest in defending the equilibrium of microecology, which was essential to establish the dominate bacteria in organs.

**Figure 3:**
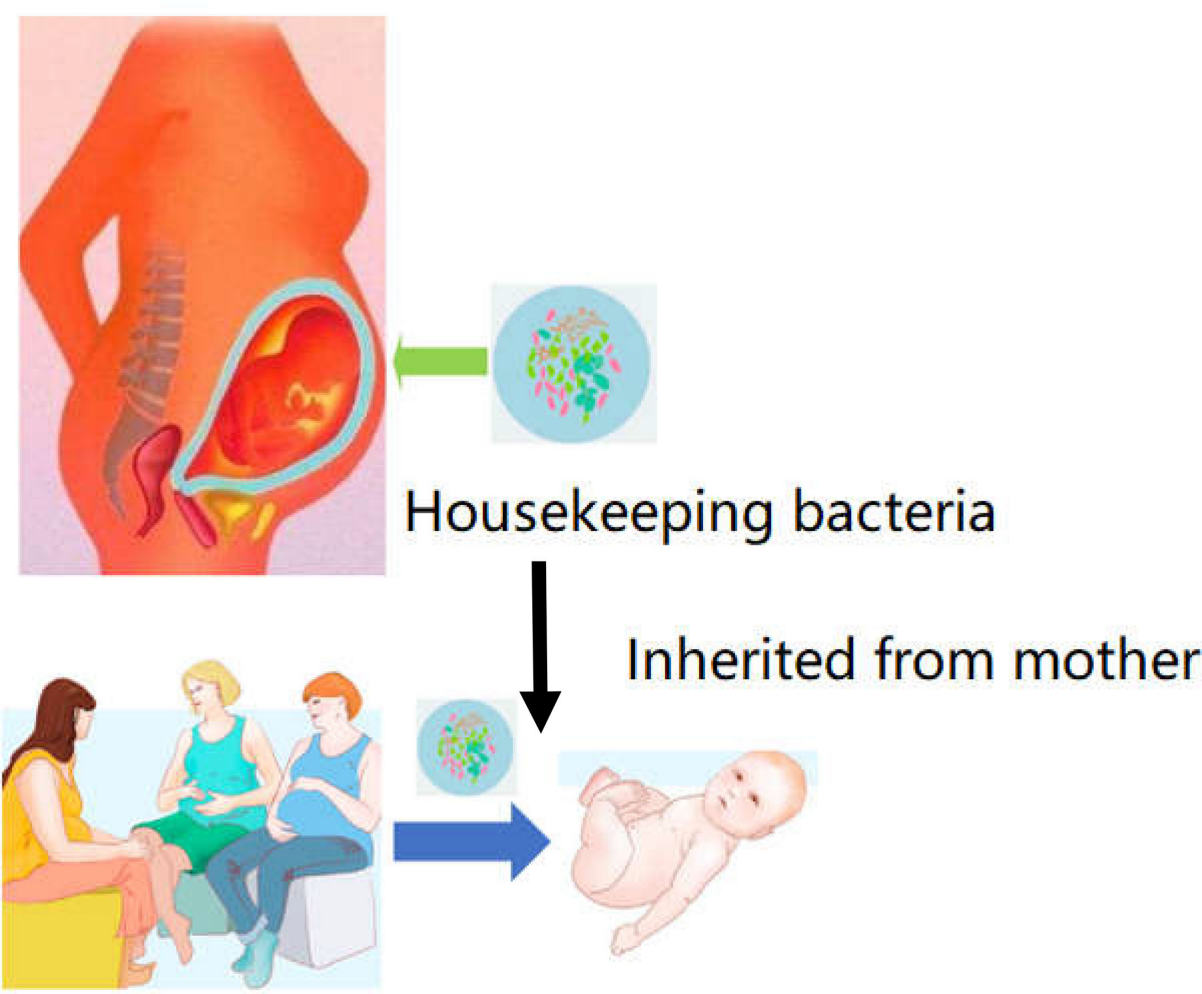
Housekeeper bacteria are the strains can be inheritable from mother to infant. Once these strains of housekeeper bacteria can be acquired from the placenta or amniotic fluid. The bacteria could be founded as a primary microbiome data to cure and prevent the dysbiosis in future GIT dysfunction.

Following the common supplementary pathway, supplementation with the scarcer bacteria of the gut is not an ideal strategy. It is hypothesized that the core bacterial community ecology (showing in **Figure 2**) plays an important role in maintaining the dynamic equilibrium of microbiota in the organs (81). Core bacteria are defined as one or several functional strains, that play important roles in maintaining the physiological wellness (82). Although the number of core bacteria is small, they serve various key functions, and several core strains are inherited in different breeds of the same animal (83). The core bacteria may also be cross-inherited from an ancestral generation or from interactions with the outer environment (84–86), shown in **Figure 3**. There is evidence that some of the microbial taxa found in the placenta, such as those in the genus *Lactobacillus*, and the common pathogen *Streptococcus agalactiae*, may have an oral origin (87–89). It is also known that vaginal microbiota mainly consists of *Lactobacillus*, namely *Lactobacillus crispatus*, *Lactobacillus iners*, and *Lactobacillus jensenii*, as well as *Gardnerella vaginalis*. The abundance and diversity of vaginal microbiota vary across different periods of pregnancy. Such data further showed that the housekeeping bacteria were part of the core bacteria can be inherited from matrix and play a key role in establishing a healthy bacterial community (2).

Certain core bacteria were commonly owned by rodent animals. In humans, using 16S rDNA cloned sequences from 17 individuals found seven genera that were common in 50% of the cohort (90). On the other hand, a study investigating metagenomes from 124 European individuals discovered that 90% of the individuals of the cohort share a common core of nine genera, at a 10% sequence coverage threshold through sequenced metagenome (91). The comparative analysis of six mouse gut microbiota datasets (92, 93) showed that the core mouse gut microbiota plateaus were partly overlapped with human.

The genera of *Lactobacillus, Akkermansia*, *Bacillus*, *Bifidobacterium*, *Clostridium* and *Prevotellacea* form the core bacteria in the GIT of humans, and their merits in the recovery from various diseases have been documented (2, 4, 94, 95). Oral administration of *Akkermansia muciniphila* has been used as a treatment to reduce the symptoms of IBD (92). Supplementation with the strains *Lactobacillus reuteri* and *Lactobacillus johnsonii* has been useful in optimizing the bacterial composition of both humans and pigs (5, 82). Addition of the strain *Clostridium propionate* helped to improve body health and overcomes stress (93). Co-correlation network analysis revealed that the genus of *Prevotellacea UCG-003* was the key bacterium in microbiota of piglets. Furthermore, changes in bacterial metabolic function between diarrheic piglets and non-diarrheic piglets were estimated by picrust analysis (contained in Metagenomic analysis), which revealed that the dominant functions of fecal microbes were membrane transport, carbohydrate metabolism, amino acid metabolism, and energy metabolism. Also, the 16S rDNA cloned sequence on colostrum of cows found that ten core genera contained *Bacillus*, *Bacteroides*, *Staphylococcus*, *Acinetobacter*, and *Pseudomonas.* A part of the found core bacteria belonged to the common intestinal bacteria and were also identified as the core ones.

### 5.2 Established the domination of core bacteria

**Figure 4:**
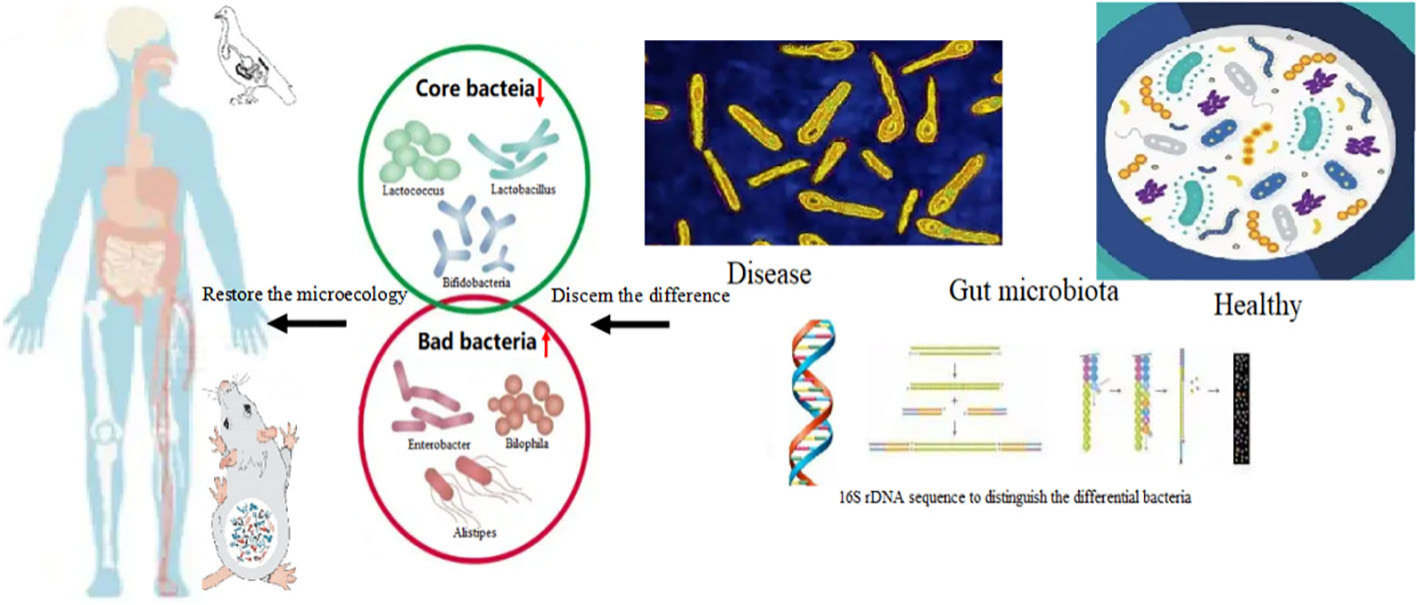
The core bacteria in diseases and restore the microbiota first supplementation with the core bacteria. The core bacteria such as the genus of *Lactobacillus*, *Akkermansia, Bifidobacterium*, the abundance was depleted in gut bacteria of patient with obesity and gout. The abnormal increasing of the bacteria namely, *Enterobacter, Alistipes*, *Bilophila*, and *Bacteroides* can be detected. Supplementation with core bacteria such as *Lactobacillus reuteri*, *Lactobacillus salivarius*, *Akkermansia muciniphila Bifidobacteria infantis*, *Bifidobacteria longum* and *Bifidobacteria animalis, Faecalibacterium prausnitzii*, restore the dominant flora, and repair the equilibrium of microecology. Storage of core bacterial database in health condition to establish a unique individual sets is necessary for self-aid for whom in disease. The cases in animal model such as poultry and mouse were also found the core bacteria supplementation could alleviate the symptoms. Chicks fed with high crude protein diet induced gout, and supplementation with *Lactobacillus reuteri* can restore the bacterial composition. Through comparing the differences of bacterial composition of health and diseased conditions, get the common useful strains of core bacteria through culture-independent metagenomic approaches is essential, which would be achieve the targeted supplementation of bacteria.

Gut microbial diversity and the dominate microbiota changed in disease. It is assumed that the core strains of bacteria play more direct roles in assisting the receptors to restore bodily health (96). From these studies, it can be concluded that the core strains of bacteria should be further identified and their functions in different stages of growth should be determined (97). Replaced with bacteria at the site of microbial translocation by replacement with the core bacterial strains, following supplementation, can be done based on the relationship between the disease and bacterial composition (98). Restore the dominant flora, and repair the equilibrium of microecology (**Figure 4**). The supplementation of core bacteria is essential to promote resilience of bacterial abundances. The theory of dominant flora in microbiology suggested that the core bacteria inherited form ancestors dominated in all strains of bacteria in gastrointestinal tract. More than 1700 strains of bacteria found in obese volunteers and these accept the dietary intervention with high fibers, suggested that the total of 141 strains of bacteria composed 2 different sets, with one set increased and the other decreased. Otherwise, the situation was reverse after dietary intervention. Nearly, 50 strains of bacteria were composed core bacteria increased after dietary intervention and suppress the remained strains. The 141 strains of bacteria covered less than 10% total bacteria in GIT. Others were owing no ecological network in micro-system (99–101).

Supplementation with the reduced abundance of fiber fermenting bacterial strains such as *Lactobacillus acidophilus*, *Lactobacillus casei*, *Lactococcus lactis*, *Bifidobacterium bifidum*, and *Bifidobacterium lactis* were helpful in obese patients (102), which can reduce the abundance of *Desulfovibrio* species and increase in the abundance of *Clostridium* species, are key features restore the dominate bacteria in obesity model and are seen in humans with age-associated metabolic syndrome (81). Cases in animal model such as poultry and mouse were also found the core bacteria supplementation could alleviate the symptoms. Chicks fed with high crude protein diet induced gout, and supplementation with *Lactobacillus reuteri* can restore the bacterial composition (99, 103).

By identifying the differences in bacterial composition associated with different diseases, microbiota transplantation can be tailored to supplement the deficient bacterial genera. In unhealthy individuals, outnumbered and short-numbered microbiota can be identified through meta-genome sequencing (104) after which the dominant genera of bacteria in the healthy status must be defined (105, 106). The goal of such research is to develop powerful probiotic regimens that can replace FMT.

However, there is still a lack of sufficient meta-data to distinguish bacterial differences in patients with different disease currently. Storage of core bacterial database in health condition to establish a unique individual sets is necessary for self-aid for whom in disease. Differences in the microbiota profile have been reported to be related to known clinical risk factors for many diseases, such as metabolic diseases, asthma, arthritis, and cancer. In this sense, personalized probiotic therapies aiming to manipulate the host microbiota using specific and different strains have gained considerable interest in recent years. If we continue to learn about other gut microorganisms and their roles in human health, we may obtain a complete rationale for selecting the next generation of probiotics, such as *Clostridia* clusters IV, XIVa and, XVIII and *Faecalibacterium prausnitzii*, which have emerged as non-traditional probiotics and studies on their effects on inflammatory diseases have been met with promising results. *F*. *prausnitzii* is a commensal bacterial strain that has been reported to be less abundant in colitis patients. However, *F*. *prausnitzii* exhibited an important anti-inflammatory response in mouse experimental colitis models. Most of these effects were linked to the high capacity of this strain to produce metabolites with anti-inflammatory effects. *Clostridium butyricum* is another potential non-traditional candidate probiotic that can produce metabolites with anti-inflammatory effects, such as butyrate (30, 107).

With the development of sequencing technology, microbiome data such as strain-level variation, transcriptomics, proteomics, and metabolomics (108), can be determined. Future avenues and challenges for understanding the interplay between human nutrition, genetics, and microbial genetics, need to be addressed. In addition, integrating microbiome data with human multi-omics data, such as genetics, transcriptomics, epigenetics, and metabolomics, should be considered to develop successful treatments in the future.

In the treatment of these diseases, beneficial microbial functioning decreases, thus, supplementation with core functional probiotics can restore the resilience of the microbial community (5). Second, identification of suitable core probiotics to inhibit the growth of specific pathogenic bacteria in patients seems to be an effective strategy to control deterioration in health caused by disease (65, 109).

### 5.3 Strategies of probiotic isolation and selection for supplementation

#### 5.3.1 Metagenomic and comparative genomics

Culture-independent metagenomic approaches to characterize the microbiota, enabled by next-generation sequencing, have increased the sensitivity and power of such associative studies by enabling high throughput analysis. Microbiota in dysbiosis-mediate diseases could be analyzed and compared with health condition in many cases of illness (110–114). Such culture independent methods of bacterial species analysis where >99% of microbial species can be unculturable (81). Taxonomic diversity can also be determined from shotgun sequenced metagenomics data sets (84, 85).

The storage of core bacterial database in health condition to establish a unique individual sets is necessary for self-aid for whom in disease. Also, compare the differences between donors or animals to conduct the common useful strains of core bacteria through culture-independent metagenomic approaches is essential (**Figure 4**). Acknowledged that core bacteria and dominant bacteria, use culture-dependent approaches to acquire the needed bacteria to supplement become urgent. The strategies for targeted choice of probiotic bacteria were suggested.

#### 5.3.2 Homogenous origin

**Figure 5:**
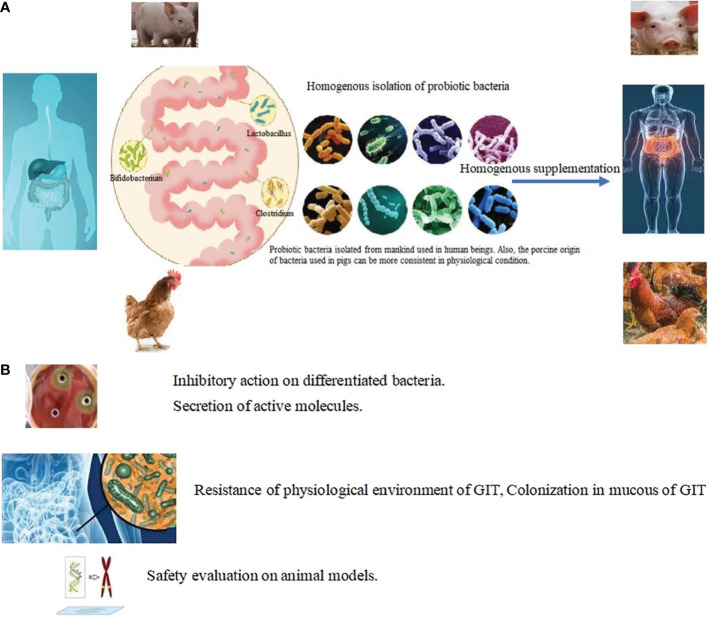
Targeted isolation in choosing probiotic bacteria. **(A)** Homogeneous isolation: Probiotic bacteria isolated from mankind used in human beings. Also, the porcine origin of bacteria used in pigs can be more consistent in physiological condition. *Lactobacillus, Akkermansia*, *Bacillus*, *Bifidobacterium*, *Lactococcus*, *Streptococcus*, *Clostridium* and *Prevotellacea* were harvested from organs and supplemented homogenously in same animals. **(B)** Targeted methods in choosing probiotic bacteria.

In restoring a healthy microbial composition, the supplementary bacteria must be ascertained to mine the probiotic strains needed to achieve precise supplementation. Currently, the most frequently used genus of bacteria in human and animal probiotic products are *Bacillus*, *Lactobacillus*, *Bifidobacterium*, and *Clostridium* (115). However, the origin of the strains being used is diverse. Many candidate strains of bacteria can be harvested through various isolation methods (116–118). The strains can be further narrowed down after certain conditional settings. In different animals, the isolation conditions of the core bacteria can be diverse (94, 119–121). Homogeneous isolation involves origination of the probiotic bacterial strains from the organs of used animals (**Figure 5A**). There are slight differences between the organs in different animal species (122, 123) and the core probiotic strains do not correspond with each other. Thus, the adaption of using bacteria colonized in the organs of animals could be more optimal than the use of non-homogeneous strains of bacteria (124). The mechanism of action is mainly related to the digestive enzymes and their roles in the gastrointestinal microenvironment. For *Bacillus* isolated from chicken, homologous feeding of probiotic bacteria for the same breed of chick could play more beneficial roles than non-homologous feeding (89, 125). Targeted supplementation in animals also considers the regular pattern of microbial development (126). In addition, supplementation with scarce strains of bacteria seems to be a limiting factor in disease recovery. The growth stage and health status should be considered as additional elimination conditions during isolation of bacteria.

#### 5.3.3 Screening conditions

Through selective culturable medium and culturing conditions, the target genus of bacteria could be isolated, such as isolation of *Lactobacilli* grown in de Man, Rogosa, and Sharpe medium, the use of BBL Trypticase soy agar with sheep blood (TSA II) broth medium for *Bifidobacteria* isolation, and M17 broth for culturing probiotic lactic *streptococci* (127).

High temperatures such as 80 °C can be used to isolate heat resistance bacteria and the oxygen content used in the culturing conditions can be adjusted to narrow the scope of isolated bacteria. Other assays of beneficial factors produced by bacteria have also proven useful in minimizing the candidate strains. These include inhibition of pathogens or conditional pathogens (128, 129); secretion of digestive enzymes, lactic acid, and digestive enzymes (130–133), the production of bioactive peptides, and vitamins such as bacteriocins and vitamin B_6_, K, and gamma amino butyric acid. The ability to perform bile salt degradation, cholesterol lysis (89, 134) and tolerance bile salts, gastric acid, and heat stress (116, 135, 135) are often used as elimination conditions (**Figure 5B**). Through a series of isolation methods, two or three strains of bacteria can finally be identified for clinical use (136–138). Details of various strategies for isolation and characterization including adhesion and colonization in the mucous membrane of the GIT (27) and safety assessments (139, 140) such as oral acute toxicity, sub-chronic toxicity, chronic toxicity, and reproductive and developmental toxicity (140, 141); antibiotic resistance; and lack of DNase (95, 139), gelatinase, and hemolytic activity (142) are shown in **Table 2**. Through a series of target conditional choice, the remained strains can be used to monitor the supplementary effect.

**Table 2:** Strategy of the isolating measurement.

Targeting strategies	Assays	References
Nutrition utilization	selective culturable medium	124;
cultured condition	High temperature, different oxygen content	143; 59
Inhibitory action on food-borne pathogenic bacteria: *Staphylococcus aureus*, *Escherichia coli*, *Klebsiella pneumoniae*, *Clostridium difficile* or *Salmonella typhi*	Inhibitory assays: Plate methods, Oxford cup method, Co-culture of probiotic bacteria and pathogen.	24, 123, 124
Secretion of digestive enzymes, lactic acid, bioactive peptides, vitamins	Plate methods on nutritional substances lysis, High performance liquid chromatography	89, 125, 126; 144
Lysis of cholesterol	Enzymatic lysis assay *in vitro*.	128
Tolerance of bile salt	Plate live bacteria counting methods	129
Tolerance of gastric acidic		111;
Tolerance of heat		130;
Tolerance of digestive enzymes in GIT		130;
Adhesion in mucous membranes of GIT	Fluorescence *in situ* hybridization	6, 78
Safety Assessment	Oral toxicity of animal model (mouse)	134; 140
Toxicity Pathogenicity/toxicogenicity	Glucose concentration, glutamic-oxalacetic transaminase activity, C-reactive protein	135, 146
Reproductive and developmental toxicity	Animal model testing	145
Antibiotic resistance, lack of DNase, gelatinase, and hemolytic activity	Meta-genome sequencing	101, 134

#### 5.3.4 Other omics technologies

While the dominant strains of bacteria play a crucial role in overall bodily health, it is also important to isolate undiscovered strains and study their functions, to aid in targeted isolation (147–150). Some bacteria are still difficult to isolate due to being unculturable in aerobic conditions (151–153). For culturable bacteria, there are abundant strains whose physiological characters can be studied to determine their benefits to the body (154, 155). Meanwhile, progress in culture techniques along with high-resolution mass spectrometry have provided an additional sight through which to identify small molecules correlated with disease, setting new direction for more rigorous studies. Multi-omic technologies, such as metagenomics, metabolomics, and culturomics, should be applied in combination to further explore core strains of probiotic bacteria. Culture-independent metagenomic approaches have increased the sensitivity and power of such associative studies by enabling high-throughput analysis (156). By applying shotgun metagenomic sequencing, the bacterial species present in the original fecal samples that grew as distinct colonies under different culture conditions can be compared and profiled. When compared with a comprehensive gene catalogue that was derived from culture-independent methods used to assess the intestinal microbiota of normal healthy individuals, identified genes in the larger database were represented, and derived metagenomic species were detected in the cultured samples (148, 157, 158). Through these methods an increasing number of unidentified microorganisms can be cultured and recognized.

By harvesting the targeted probiotic bacteria, the whole genome can be sequenced and blasted to ascertain the function employed in matrix assisted laser desorption ionization (MALDI) to identify the unknown bacteria in the database.

Although the omics technologies have improved the capacity of bacterial analysis and culture. A total of less than 1% bacteria can be successfully cultured *in vitro*, a greater number of core bacteria and cross the animals still need to further unveiled to help patient reconstruct the health microbiological flora.

## 6 Conclusion

Considering the causal relationship on intestinal bacteria and disease, supplementary probiotics to restore the bacterial composition to cure disease is performed in human and animals. In their clinical use, probiotic bacteria were not conforming with the differential scared bacteria owning the symptom, which functioned in healthy condition. Supplemented with core bacteria to re-construct the dominant flora and repair the differential bacteria was the rule. Then repair the conditioned and supper growth-ed bacteria to restore the intestinal bacteria. Skills in choice of bacteria, such as isolated from homogenous animal, conditioned with colonization, temperature, inhibition on certain pathogen, and others could minimize the candidate strains should be focused. Also, research on metagenomics and culturomics can further help to unveil the microbial composition in the organs. Strategies for targeted selection of strains directly corresponding to the deficiencies can be used to tailor precise probiotic bacterial supplementation. Considering the core bacteria in regulation bodily function, get the personal bacterial database in young and health period and make the personal tabs to distinguish the disabled conditions, which can be efficient to achieve targeted supplementation. Our review provided a venue on precise supplementation of probiotic bacteria in clinical use.

## Author contributions

JY carried out the literature study and JY drafted the manuscript. SQ critically evaluated the manuscript. HZ reviewed the manuscript. All authors approved the final manuscript. All authors contributed to the article and approved the submitted version.

## Funding

This work was supported by fund of Tibet Major Science and Technology Project (XZ202101ZD0005N), Start-up for Scientific Research of High-level Talents of Jiangsu Vocational College of Agriculture and Forestry, Youth support project of Jiangsu Vocational College of Agriculture and Forestry (2021kj19), China Agriculture Research System (CARS-40), Key projects of Natural Science Foundation of Tianjin (20JCZDJC00170).

## Conflict of interest

The authors declare that the research was conducted in the absence of any commercial or financial relationships that could be construed as a potential conflict of interest.

## Publisher’s note

All claims expressed in this article are solely those of the authors and do not necessarily represent those of their affiliated organizations, or those of the publisher, the editors and the reviewers. Any product that may be evaluated in this article, or claim that may be made by its manufacturer, is not guaranteed or endorsed by the publisher.
